# The Design of a Novel Alkali-Activated Binder for Solidifying Silty Soft Clay and the Study of Its Solidification Mechanism

**DOI:** 10.3390/ma17102177

**Published:** 2024-05-07

**Authors:** Yaohui Jing, Yannian Zhang, Lin Zhang, Qingjie Wang

**Affiliations:** 1 School of Civil Engineering, Jilin University of Architecture and Technology, Changchun 130114, China; jingyaohui0221@163.com (Y.J.); zyntiger@163.com (Y.Z.); 2 School of Civil Engineering, Dalian Jiaotong University, Dalian 116028, China; 3 School of Civil Engineering, Shenyang Jianzhu University, Shenyang 110168, China; wangqingjie01@163.com

**Keywords:** alkali-activated binder, silty soft clay, unconfined compressive strength, solidification mechanism, additives

## Abstract

In order to overcome the problems of the high economic and environmental costs of a traditional ordinary portland cement-based binder, this study used self-combusted coal gangue (SCCG), granulated blast furnace slag (GBFS) and phosphorous slag (PS) to prepare a novel SCCG-GBFS-PS (SGP) ternary alkali-activated binder for solidifying silty soft clay (SC). Firstly, the parameters of the SGP ternary binder were optimized using orthogonal experiments. Then the effects of the SGP ternary binder content (mass ratio of the SGP ternary binder and the SGP-solidified soil), initial water content of SC (mass ratio of SC’ water and SC) and types of additives on the unconfined compressive strength (UCS) of the SGP-solidified soil were analyzed. Finally, the hydration products and microstructure of the SGP-solidified soil were analyzed to investigate the solidification mechanism of the SGP ternary binder. The results showed that the optimal mass ratio of GBFS and PS is 2:1, and the optimal alkali activator content (mass ratio of Na_2_O and the SGP ternary binder) and modulus of alkali activator (molar ratio of SiO_2_ and Na_2_O of alkali activator) were 13% and 1.3, respectively. When the SGP ternary binder content was 16% and the initial water content of SC was 35%, the SGP-solidified soil met the requirement of UCS for tertiary cured soil. The incorporation of triethanolamine and polyvinyl alcohol improved the UCS, while the incorporation of Na_2_SO_4_ significantly deteriorated the UCS of the SGP-solidified soil. The C-S-H gels and C(N)-A-S-H gels generated by hydration of the SGP-solidified soil were interspersed, interwoven and adhered to each other to form a network-like space structure that played the roles of skeleton, bonding soil particles and filling pores, which improved the macroscopic properties of the SGP-solidified soil. The results of this study provide a reference for the design and development of a solid waste-based binder for solidifying SC.

## 1. Introduction

Riverside silty soft clay (SC) has difficulty satisfying construction requirements due to its high water content, relatively large pores, high compressibility and poor bearing capacity [1,2,3]. Therefore, it is particularly important to solidify SC quickly and efficiently so as to improve these properties [4,5]. Benefiting from the advantages of high in situ clay utilization, low environmental impact and adaptability, a binder is widely used for solidifying SC [6]. Ordinary portland cement (OPC) and lime are commonly used in the preparation of the binder to improve the properties of problematic soils [7]. The OPC-based binder is capable of undergoing a series of physico-chemical interactions with SC to optimize the properties of solidified soils [8,9,10]. However, the production process of OPC is energy-intensive and emits large amounts of pollutants as well as greenhouse gases, which poses a huge environmental burden [11,12]. There is an urgent need to develop an environmentally friendly binder with excellent performance to replace OPC.

The alkali-activated binder consists of an alkali activator, precursors and auxiliary materials. Precursor particles, mainly calcined natural minerals (e.g., metakaolin (MK)) or industrial solid wastes (e.g., granulated blast furnace slag (GBFS) and fly ash (FA)), contain reactive silica–alumina oxides that can be stimulated by alkali activators to produce C-S-H and C(N)-A-S-H gels through a series of dissolution–diffusion–polymerization reactions [13,14]. These gels are able to connect the soil particles and fill the pores to improve the microstructure of SC, thus improving the properties of SC [13,15]. Furthermore, the alkali-activated binder reduces carbon emissions by 80% during production compared to the traditional OPC-based binder [16]. Researchers are gradually focusing on the use of the alkali-activated binder as an alternative to OPC for solidifying SC. Lang et al. [17] explored the use of an alkali-activated binder instead of OPC for solidifying dredged sludge (DS) with different water contents. The results of their study showed that the change in water content significantly affected the unconfined compressive strength (UCS) of the solidified DS. Composite activators were more effective than single activators in activating GBFS to achieve a higher UCS of solidified DS. Murmu et al. [18] investigated the feasibility of solidifying black cotton soil (BCS) using FA geopolymer. The results showed that FA geopolymer exhibited a good solidifying effect on BCS, even at a lower alkali solution concentration (5 M sodium hydroxide). Zhang et al. [19] verified the feasibility of MK-based geopolymer as a binder for solidifying SC from multiple perspectives. The results indicated that with increasing geopolymer concentrations, the compressive strength, failure strain and Young’s modulus of the solidifying SC specimens increased, and shrinkage strains during curing decreased. Miraki et al. [20] investigated the potential of using an alkali-activated GBFS-volcanic ash (VA) binder for solidifying SC from multiple perspectives. The results showed that the combination of VA and GBFS provided sufficient calcium, silicon and aluminum to promote the formation of N-A-S-H and C-(A)-S-H gels. The incorporation of GBFS resulted in remarkably superior resistance against wet–dry and freeze–thaw cycles, as well as low carbon footprints. Chen et al. investigated the effect of reactive ions on the early properties of rice husk ash (RHA)-solidified soils. The results showed that the use of RHA alone could only improve the UCS of soft soils to a certain extent, but not the soaked strength of solidified soils. The UCS, shear strength and soaked strength of the solidified soils were significantly improved by modifying RHA using calcium carbide slag and MK as activators. From the above studies, it can be seen that the use of the alkali-activated binder for solidifying SC has been fruitful, but most of the studies have focused on a few materials. Broadening the pipeline of precursor materials for the preparation of an alkali-activated binder is promising [12].

Coal gangue (CG), a type of solid waste separated from coal mining and washing, is a mixture of various rocks. China’s CG annual emissions account for about 25% of all categories of industrial solid waste [21]. In 2021 alone, 743 million tons of CG were generated, of which nearly one-third spontaneously combusted under natural conditions to form self-combusted coal gangue (SCCG) [22,23]. It has been reported that the spontaneous combustion process can stimulate the activity of silicon oxide and aluminum oxide in CG to some extent [24,25]. Li et al. [24] prepared a SCCG-based alkali-activated foam with low bulk density, high total porosity, acceptable compressive strength and low thermal conductivity using the fast microwave foaming method. Liu et al. [26] compared SCCG-based/calcined CG-based geopolymer concretes, and the results showed that SCCG-based geopolymer concretes had good mechanical properties with good economic and cost benefits. Qin et al. [27] developed an OPC-CG blend paste by carbonation curing. The results showed that carbonation curing improved the compressive strength of OPC-CG pastes with a higher incorporation of CG. The specimens also showed better resistance to chloride ion penetration. The annual production of PS in China has reportedly reached 8 million tons [28]. The accumulation of PS not only leads to the waste of resources but also affects the ecological environment. Therefore, a large amount of PS is in urgent need of resource utilization. Thanks to the fact that the main chemical components of PS are SiO_2_ and Al_2_O_3_, it has the potential to be a precursor [29]. Wang et al. [30,31] systematically investigated the relationship between hydration, microstructure and compressive strength of alkali-activated PS. The results showed that the alkali activator content and modulus of the alkali activator had both positive and negative effects on PS hydration, and the overall effect depended on their relative magnitudes. The hydration of alkali-activated PS experienced a transformation of NASH first into CNASH (low Ca) and then into CNASH (high Ca) due to the stronger polarization ability of Ca^2+^ over Na^+^, and the gel transformation was accompanied by dealumination. Yang et al. [28] found that the poor performance of the PS-based geopolymer was due to its aluminum deficiency. The results also indicated that ultrafine FA and more activators contributed to the Al and high alkalinity environments, which positively induced the production of more geopolymer gels, thus releasing more heat and optimizing the pore structure of the matrix. A similar conclusion was reached in the study of Zhang et al. [32]. PS mixed with GBFS to prepare alkali-activated composite cementitious materials can overcome the problem of the poor early compressive strength of PS-based geopolymers.

This literature review demonstrates that the utilization of the SCCG/PS-based geopolymer is mainly focused on construction. Relatively few studies have been conducted on the preparation of an alkali-activated binder using SCCG or PS for application in solidifying SC. Based on this, the aim of this paper is to investigate the feasibility of developing a SCCG-GBFS-PS (SGP) alkali-activated ternary binder using SCCG, GBFS and PS for solidifying SC. The range analysis of the orthogonal experiment was first used to optimize the alkali activator content, modulus of the alkali activator and mass ratio of the GBFS and PS of the SGP ternary binder. The effects of the SGP ternary binder content, initial water content of SC and types of additives on the 7 d and 28 d USC of solidified soils were subsequently explored. Finally, the solidifying mechanism of the SGP-solidified soil was analyzed by conducting microstructural property analysis.

## 2. Materials and Methods

### 2.1. Materials

#### 2.1.1. Silty Soft Clay Sample

**Figure 1:**
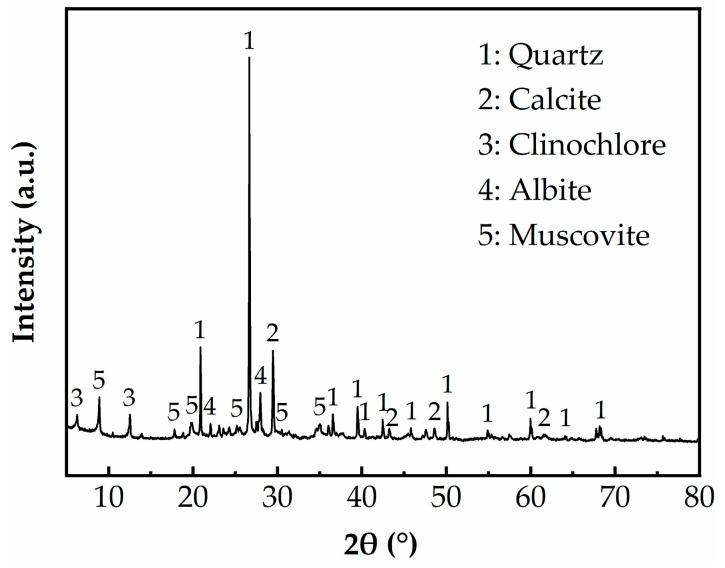
XRD pattern of SC.

The untreated SC was taken from the bank of a river in Liaocheng city (Shandong Province, China) at a depth of 2–3.0 m. The SC was yellowish-brown, softly clayey, and had great compressibility. According to the requirements of the “highway geotechnical test” (JTG 3430-2020) [3], the water content, liquid–plastic limit and other relevant physical indexes of SC were measured. From Table 1, it can be seen that SC had a higher water content of 47%, a liquid limit of W_L_ = 41.9%, a plastic limit of W_P_ = 22.3%, a plasticity index of I_P_ = 19.6 and a specific gravity of 2.60 g/cm^3^. The mineral phase composition of the SC was analyzed by an X-ray diffractometer (XRD) (Bruker D8 Advance, Bremen, Germany). As shown in Figure 1, the mineral phase components of SC were mainly quartz, calcite, clinochlore, albite and muscovite.

#### 2.1.2. SGP Ternary Binder

**Figure 2:**
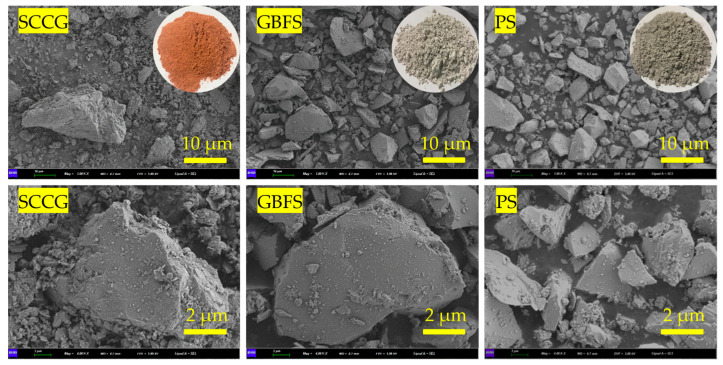
The microstructure images of raw materials.

**Figure 3:**
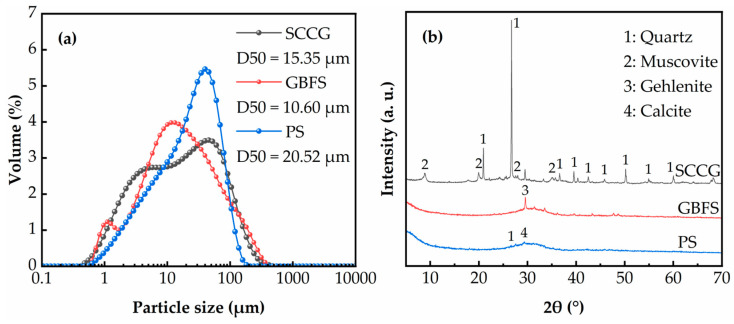
(**a**) Particle size distribution of raw materials; (**b**) XRD patterns of raw materials.

The SGP ternary binder was prepared using SCCG, GBFS and PS as ternary precursors, where SCCG and PS needed to be used after grinding for 15 min using a planetary ball mill (Tencan Powder XQM-4, Changsha, China). The SCCG was taken from a coal gangue mountain in Chaoyang City (Liaoning Province, China). From Figure 2, it can be seen that the SCCG had a red morphology, and its obvious laminated structure can be observed after magnification using a scanning electron microscope (SEM) (ZEISS GeminiSEM 300, Jena, Germany). S105-grade GBFS was purchased from a building materials company in Gongyi City (Henan Province, China). It can be seen from Figure 2 that the GBFS particles were milky white, and continued magnification revealed that the GBFS had a smooth, plate-like structure. PS was supplied by Yunnan Kunming Haifu Trading Co., Ltd. (Kunming, Yunnan Province, China). From Figure 2, it can be seen that PS was brown, and continued magnification revealed that PS had an irregular lumpy structure. The specific surface areas of SCCG, GBFS and PS were measured as 1110 m^2^/kg, 1206 m^2^/kg and 724 m^2^/kg, respectively. The particle size distributions of the three raw materials were measured using a laser particle sizer (Malvern Mastersizer 3000, London, UK), and the results are shown in Figure 3a. The mineral phase compositions of the three raw materials were analyzed using XRD, and it can be seen from Figure 3b that the mineral phase composition of SCCG was mainly quartz and albite. GBFS had an obvious hump between 20° and 40°, which indicated a very high glassy phase composition, and its main mineral phase composition was mainly gehlenite [33]. The mineral phase composition of PS was mainly quartz and calcite, and it also had an obvious hump, but the area of the hump was smaller than that of GBFS. The chemical composition of the three raw materials as well as SC was analyzed using an X-ray fluorescence spectrometer (XRF) (Panalytical Axios, Almelo, The Netherlands), and the results are shown in Table 2.

The alkali activator solution was prepared with deionized water, sodium silicate and sodium hydroxide. Sodium silicate was purchased from Porun Refractories Co., Ltd. (Zhengzhou, Henan Province, China) with an initial modulus (molar ratio of SiO_2_ and Na_2_O) of 2. Sodium hydroxide was supplied by a building materials company in Tianjin. The modulus of the alkali activator was adjusted by adding sodium hydroxide to the sodium silicate solution. In addition, alkali activator solutions of the appropriate modulus needed to be prepared 24 h in advance for adequate cooling [34,35,36].

### 2.2. Mix Proportion Preparation

The mixing of the SGP-solidified soil was carried out with reference to the “Mix Proportion Design Of Cement Soil (JGJ/T 233-2011)” [37]. The orthogonal experiments were conducted to investigate the effects of the alkali activator content (mass ratio of Na_2_O and the SGP ternary binder), the modulus of the alkali activator (molar ratio of SiO_2_ and Na_2_O) and the mass ratio of GBFS and PS of the SGP ternary binder (fixed SCCG internal dosing ratio of 50%) on the USC of the SGP-solidified soil, respectively. An L9(3^3^) orthogonal experiment was designed to analyze these factors in an optimized sequence. The factors and levels of orthogonal experiments are illustrated in Table 3. Then the effect of the SGP ternary binder content (mass ratio of the SGP ternary binder and the SGP-solidified soil) (Series D), initial water content of SC (mass ratio of SC’ water and SC) (Series W) and types of additives (Series F) on the 7 d and 28 d USC were explored, which were designed as shown in Table 4. Finally, the solidification mechanism of the SGP ternary binder on SC was analyzed by XRD and SEM–energy dispersive spectrometer (EDS).

**Figure 4:**
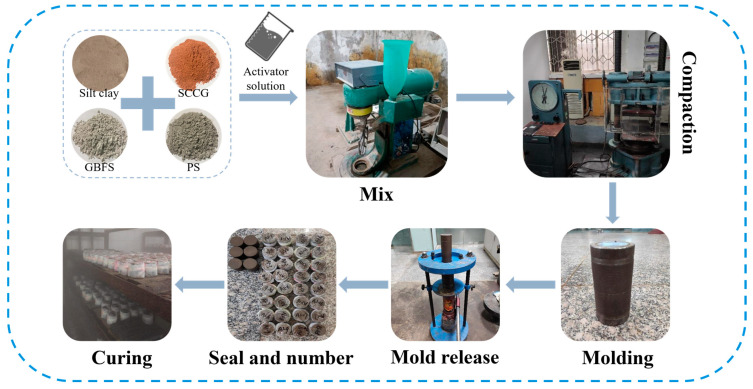
Flow chart for the preparation of the SGP-solidified soil.

Molding requires mixing the SGP-solidified soil into the 50 mm × 50 mm vaseline-coated cylindrical steel film three times; each layer of the SGP-solidified soil needed to be pounded, the surface scraped and then loaded into the next layer of the SGP-solidified soil until the soil sample was filled to the top of the mold at a height of 2 cm from the exposed pads, the surface scraped flat and put in the top pads. The prepared SGP-solidified soil was then de-molded by static pressure molding using a press with a loading rate of 2 mm/min, and then the specimens were numbered, sealed with plastic wrap and placed in a standard maintenance room for maintenance until the required age to complete the required experiments. The specific preparation process of the SGP-solidified soil is shown in Figure 4.

### 2.3. Testing and Characterization

#### 2.3.1. Unconfined Compression Strength Test (UCS)

The UCS of the SGP-solidified soil was determined in accordance with the “Standard for Geotechnical Testing Methods” (GB/T50123-2019) [38]. A CMT5105-type SANS microcomputer-controlled electronic universal testing machine was used to test the UCS of the SGP-solidified soil at a loading rate of 1 mm/min. Three samples in each group were tested in parallel, and the average value was recorded as the unconfined compressive strength. When the strength of the specimen differed from the average value by more than 10%, it was adjusted, and the average value was taken as the value of the test strength by re-producing the specimen.

#### 2.3.2. Microstructural Property Analysis

The specimens cured to the specified age were soaked in anhydrous ethanol for 3 days to terminate hydration. After the completion of the soaking, they were naturally dried for 6 h. The dried specimens were then ground and passed through a 200-mesh sieve. Finally, XRD was used to analyze the evolution of the physical phases of the SGP-solidified soil under different factors. SEM equipped with an EDS was used to analyze the microscopic morphology of the specimens, in which the specimens were required to retain a block structure with a size of 1 cm × 1 cm × 0.5 cm.

## 3. Results and Discussion

### 3.1. Orthogonal Experiment Analysis

**Figure 5:**
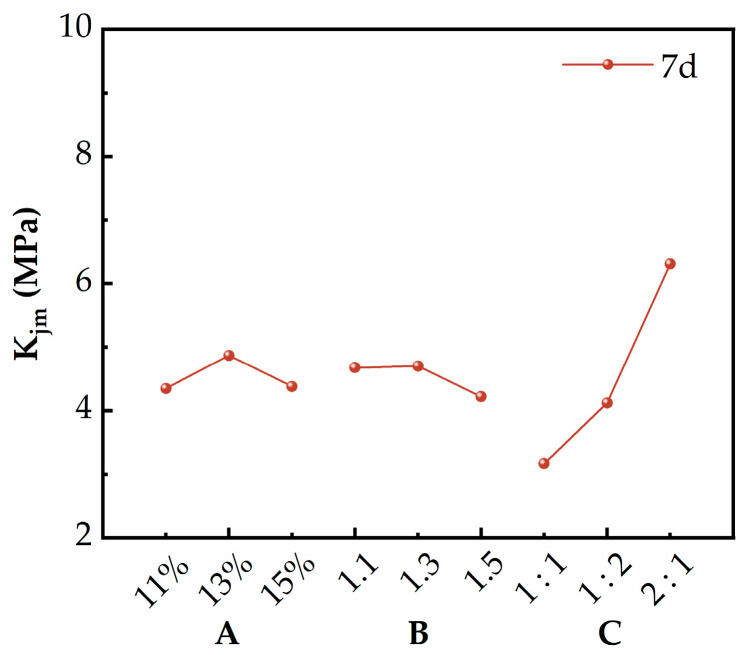
The influence of various factors.

The average 7 d UCS results of the SGP-solidified soil are shown in Table 5. Referring to the literature [2,3,39], the range analysis method was used to analyze the data in Table 5 to determine the optimal level of each factor and the optimal combination. The formula for calculating the range is “R_j_ = [max (K_j1_, K_j2_, K_j3_) − min (K_j1_, K_j2_, K_j3_)]/3”. K_jm_ is the sum of the test indexes corresponding to the m-th level of the factors in the j-th column; R_j_ is the range of the factors in the j-th column, indicating the change range of the indexes. R_j_ can be used as a measure to compare the degree of influence of each factor on the UCS. When the value of R_j_ is large, it indicates a high degree of this factor, which is usually regarded as a major factor [40,41]. As shown in Table 6, the importance order of each factor on the 7-day UCS of the SGP-solidified soil was C > A > B. As can be seen in Figure 5, the optimal level of factor A was 13%, the optimal level of factor B was 1.3 and the optimal level of factor C was GBFS:PS = 2:1. The optimal combination of factors was A_2_B_2_C_3_.

### 3.2. The Effect of the SGP Ternary Binder Content on UCS

**Figure 6:**
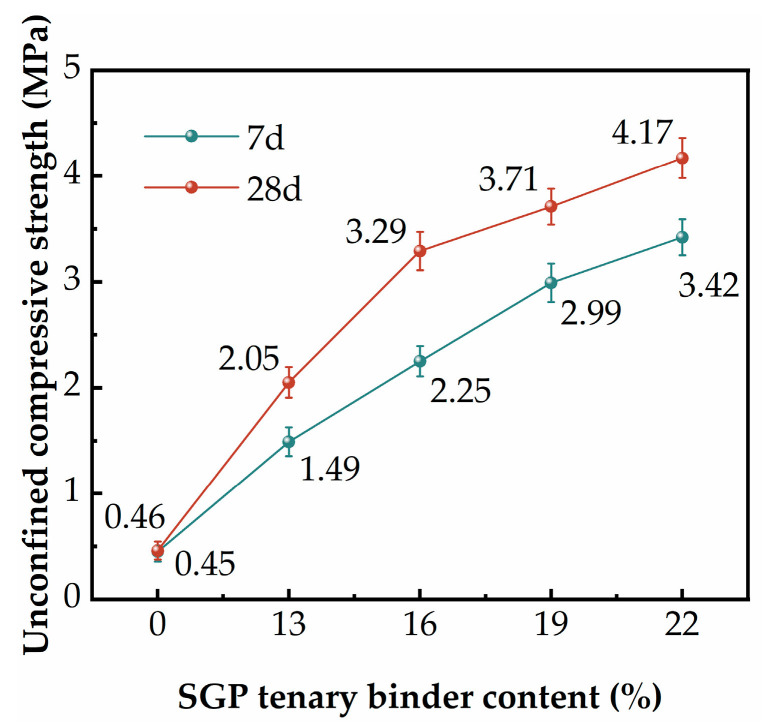
The UCS of solidified soil with different SGP ternary binder content.

Figure 6 demonstrates the effect of the SGP ternary binder content on the UCS of the SGP-solidified soil. When the content of the SGP ternary binder was 0, the 7 d and 28 d UCS of the SGP-solidified soil were very low and basically remained unchanged, which could not meet the needs of practical engineering. With the increase in the SGP ternary binder content, the UCS of the SGP-solidified soil at the two ages had a tendency to increase. It was noteworthy that the UCS of the SGP-solidified soil increased faster when the SGP ternary binder content was less than 16%, and the growth of the SGP-solidified soil decreased after exceeding 16%. When the SGP ternary binder content was increased from 13% to 16%, the 7 d UCS of the SGP-solidified soil increased by 50% and the 28 d UCS increased by 60%. When the SGP ternary binder content was increased from 16% to 19%, the 7 d UCS of the SGP-solidified soil increased by 33% and the 28 d UCS increased by 13%. The 7 d UCS of the SGP-solidified soil increased by 15%, and the 28 d UCS increased by 12% when the SGP ternary binder content was increased from 19% to 22%. This was due to the fact that with the increase in the SGP ternary binder content, the amount of active SiO_2_ and Al_2_O_3_ increased, and the silica–oxygen and aluminum–oxygen bonds broke under the action of the alkali activator. More silica–oxygen tetrahedral and aluminum–oxygen tetrahedral were released into the system to polymerize with Ca^2+^ and Na^+^ to generate C-S-H and C(N)-A-S-H gels [42,43,44]. The gels filled the pores and bound the unhydrated particles, so that the SGP-solidified soil became denser [45]. The SGP-solidified soil mixed with the 16% SGP ternary binder met the requirements of the “Technical Standard for Application of Soil Stabilizer” (CJJ/T286-2018) [46] for the UCS of tertiary cured soil.

### 3.3. The Effect of the Initial Water Content of SC

**Figure 7:**
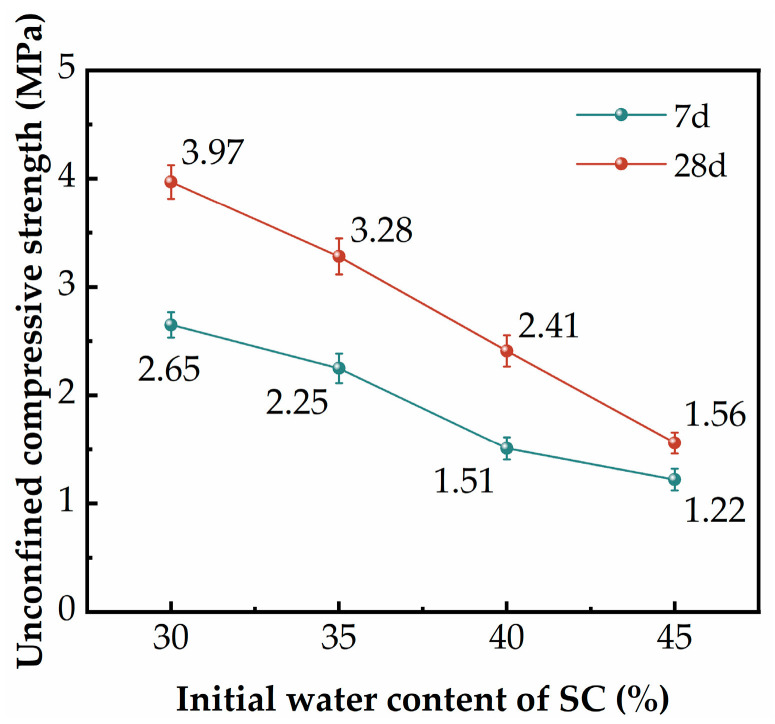
The UCS of the SGP-solidified soil with different initial water content of SC.

Figure 7 shows the effect of the initial water content of SC on the UCS of the SGP-solidified soil. As can be seen in Figure 7, the UCS of the SGP-solidified soil decreased with increasing water content. When the initial water content of SC was increased from 30% to 45%, the 7 d and 28 d UCS of the SGP-solidified soil decreased from 2.65 MPa and 3.97 MPa to 1.22 MPa and 1.56 MPa, with a decrease of about 54% and 60.8%. This was due to the fact that as the water content increased, on the one hand, the concentration of the alkaline environment in the cured soil samples decreased [47], and the amount of silica–oxygen tetrahedra and aluminum–oxygen tetrahedra dissolved by the aluminosilicates decreased, thus the amount of hydration products decreased. On the other hand, the evaporation of free water that cannot participate in the hydration reaction left pores [48], which caused the microstructure of the soil to become loose. Nevertheless, the 28 d UCS of the SGP-solidified soil was able to meet the technical requirements for the cement–soil mixing pile (>0.8 MPa, Chinese standard YBJ225-91) [49].

### 3.4. The Effect of Additives

**Figure 8:**
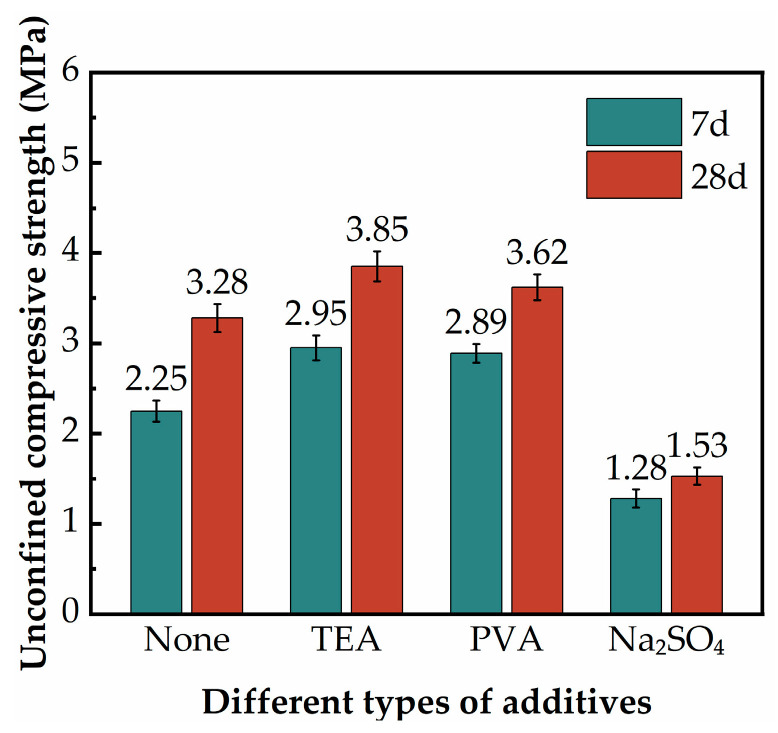
The UCS of the SGP-solidified soil with different types of additives.

Figure 8 demonstrates the effect of additive types on the UCS of the SGP-solidified soil. As shown in Figure 8, the 7 d and 28 d UCS of the SGP-solidified soil without additives were 2.25 MPa and 3.28 MPa, respectively. The 7 d and 28 d UCS of the SGP-solidified soil were 2.95 MPa and 3.85 MPa when the additive was 2% TEA, and the strengths were increased by 31% and 17%, respectively, compared with the specimens without the additive. The 7 d and 28 d UCS of the SGP-solidified soil were 2.89 MPa and 3.62 MPa, respectively, when the additive was incorporated as 2% PVA, which increased the strengths by 29% and 10% compared to the case without the additive. When the additive was 2% Na_2_SO_4_, the 7 d and 28 d UCS of the SGP-solidified soil were 1.28 MPa and 1.53 MPa, respectively, and the strengths were reduced by 43% and 53%, respectively, compared with the specimens without the additive. In summary, the strength of the SGP-solidified soil all increased with age, in which TEA and PVA had a reinforcing effect on the UCS of the SGP-solidified soil, while Na_2_SO_4_ had a weakening effect. The enhancement of PVA was due to its hydrolysis of aluminum hydroxyl complexes with large molecular weight and high charge that can play the role of electrical neutralization and adsorption bridging, strong adsorption and complexation of ions in solution, especially SiO_3_^2−^, which promoted the continued dissolution of the mineral phase components of GBFS and PS [50,51,52]. The incorporation of Na_2_SO_4_ led to a high Na^+^ concentration in the SGP-solidified soil, and the thickness of the soil particle bilayer increased after a large amount of Na^+^ was adsorbed on the surface of negatively charged soil particles [2]. The adhesion between the soil particles was weakened, which led to the microstructure of the SGP-solidified soil becoming loose. The macroscopic performance was that the UCS of the SGP-solidified soil was decreased. Among them, the optimization effect of TEA was the most obvious; therefore, the influence of different TEA dosages on the UCS of the SGP-solidified soil was investigated.

**Figure 9:**
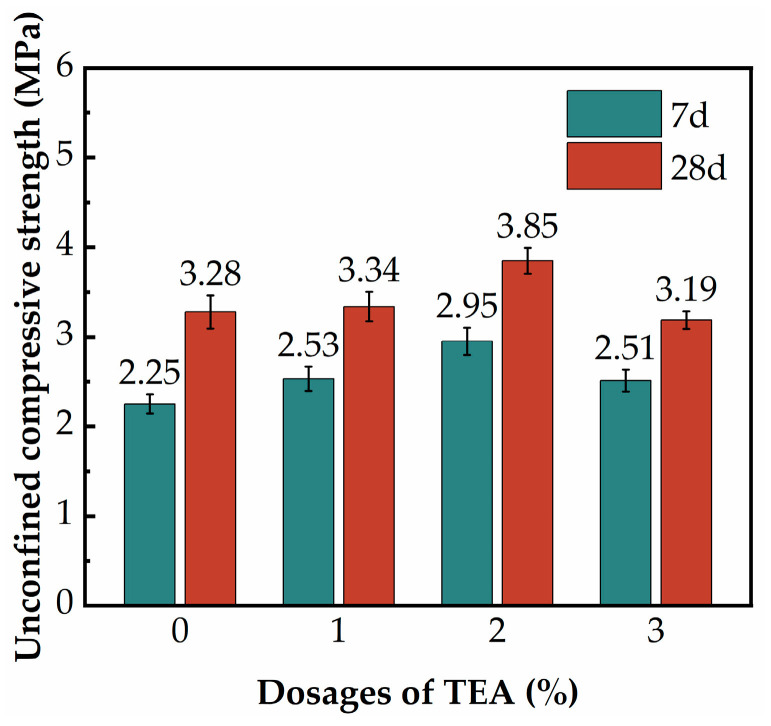
The UCS of the SGP-solidified soil with different dosages of TEA.

Figure 9 shows the effect of TEA dosages on the UCS of the SGP-solidified soil. As can be seen from Figure 9, the UCS of the SGP-solidified soil showed a tendency to first increase and then decrease with the increase in TEA dosage. The 7 d and 28 d UCS of the SGP-solidified soil without additives were 2.25 MPa and 3.28 MPa, respectively. The highest UCS of the SGP-solidified soil was reached when the TEA dosage was 2%, and the 7 d and 28 d UCS were 2.95 MPa and 3.85 MPa, and its strength increased by 31% and 17%, respectively, compared with the case without the additive. This was due to the fact that, on the one hand, TEA was a solvent with strong alkalinity, which increased the pore PH and thus promoted the breakage of silica–oxygen and aluminum–oxygen bonds in GBFS and PS, and more silica–oxygen tetrahedra and aluminum–oxygen tetrahedra were released into the system to participate in the hydration reaction. On the other hand, the nitrogen molecules in TEA had a pair of non-shared electrons, which can easily form stable complexes with metal ions, thus forming soluble zones with free water in the system, which accelerated the ion diffusion rate, promoted the polymerization of the gels and thus integrally improved the UCS of the SGP-solidified soil [53,54]. However, when the dosage of TEA was too high, the alkalinity in the pore solution of the SGP-solidified soil was too high, and the prematurely generated gels attached to the surface of the unhydrated particles [55,56], which inhibited the continued dissolution of the SGP ternary precursor particles and led to a decrease in the amount of hydration products, thus resulting in a decreasing trend in the UCS of the SGP-solidified soil.

### 3.5. Microstructural Property Analysis

#### 3.5.1. X-ray Diffraction Test Analysis

**Figure 10:**
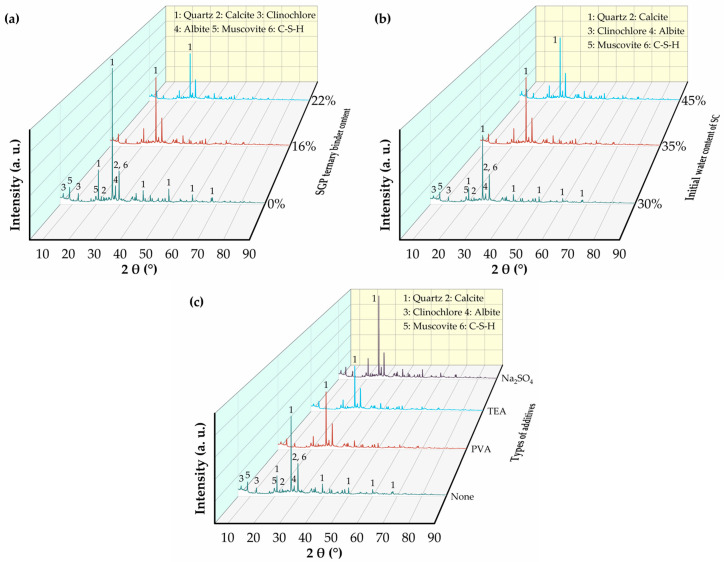
XRD patterns of the SGP-solidified soil with different (**a**) SGP ternary binder content, (**b**) initial water content of SC and (**c**) types of additives.

Figure 10 illustrates the XRD patterns of the SGP-solidified soil at different SGP ternary binder contents, initial water content of SC and types of additives. The mineralogical composition of SC was mainly quartz, dolomite, calcite and sodium feldspar. The peak shapes and positions of the curves did not change between groups of the SGP-solidified soil, indicating that the types of mineral phases of the hydration products of the SGP-solidified soil did not change with the changes in the SGP ternary binder content, the initial water content of SC and the types of additives. A “broad peak” centered at 27°–31° (2θ) was found between 20° and 40° (2θ), which suggested the coexistence of amorphous C-(A)-S-H and N-A-S-H gels [33,57,58]. In addition, although all specimens exhibited reflections of the C-S-H gels, it was difficult to identify and differentiate the peak of the C-S-H gels by XRD because of the semi-amorphous nature of the gels and the overlap with the strong diffraction peaks of calcite at around 2θ = 29° [59,60]. In summary, the hydration reaction of the SGP ternary binder belonged to the geopolymerization process, and its products were mostly amorphous and semi-amorphous substances, and the hydration products were mainly C-S-H and C(N)-A-S-H.

Figure 10a demonstrates the effect of the SGP ternary binder on the mineral phase of the SGP-solidified soil. With the increase in SGP ternary binder content from 0 to 22%, the diffraction peak intensity of quartz decreased significantly. This indicated that the incorporation of the SGP ternary binder promoted the dissolution of quartz to participate in the geopolymerization reaction, thus generating more gels. Figure 10b shows the effect of the initial water content of SC on the mineral phase composition of the SGP-solidified soil. The characteristic diffraction peaks of quartz decreased with the decrease in initial water content of SC, indicating that with the increase in initial water content of SC, the concentration of the alkaline environment in the cured soil samples decreased and the dissolution of quartz in the SGP-solidified soil decreased. Figure 10c displays the effect of additive types on the mineral phase composition of the SGP-solidified soil. The quartz diffraction peaks of the SGP-solidified soil with TEA and PVC had a decreasing trend compared with that of the unadded additive group, which indicated that TEA and PVC were able to play the roles of electro-neutralization and adsorption-bridging after dissolution. They had adsorption and complexation effects on the ions in solution, which facilitated the sustained dissolution of the quartz. However, the quartz characteristic diffraction peaks of the group adding Na_2_SO_4_ were significantly higher than those of other groups, indicating that the addition of Na_2_SO_4_ adversely hindered the hydration process of the SGP-solidified soil. This was consistent with the previous analysis of the UCS of the SGP-solidified soil.

#### 3.5.2. Micromorphology Analysis

**Figure 11:**
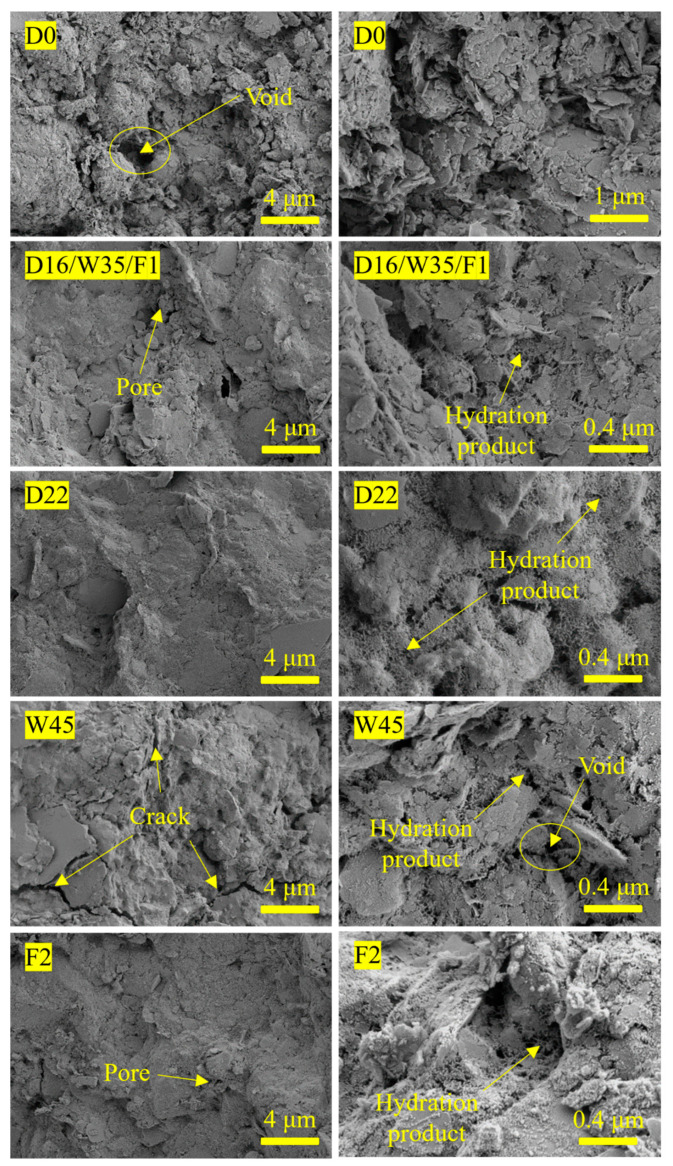
Microscopic morphology of the SGP-solidified soil at 28 days.

Figure 11 shows the microscopic morphology of the SGP-solidified soil under different influencing factors. D0 was the microscopic morphology of the SC without the SGP ternary binder at 28 days. It can be seen that the clay in the SC, which was in the form of scales, was not connected compactly, and it consisted of a large number of agglomerated block structures. The skeleton structure was relatively loose, and there were more voids and pores, which was consistent with the poor 28-day UCS of D22. Comparing D16 and D0, it can be seen that the addition of the SGP ternary binder can significantly improve the microstructure of the soil; zooming in shows a large number of gelling products filling the pore space. These gelling products were mainly formed by the reactive SiO_2_ and Al_2_O_3_ [61] in the SGP ternary binder and SC, which were dissolved in the action of the alkali activator and undergo a series of polymerization reactions with Ca^2+^ and Na^+^ to produce C(N)-A-S-H and C-S-H gels. Gels can bond soil particles, enhance the adhesion between soil particles and fill the pores to reduce porosity, so that the soil structure becomes more dense and the macro-expression of the compressive performance greatly improves. The soil structure of the SGP-solidified soil in D22 was more dense, the distance between gels was reduced and the magnification showed that the tight network-like gelling products covered the soil particles and filled the pores, which further improved the structural integrity of the SGP-solidified soil. This was consistent with the better 28-day UCS of D22.

Comparison of W45 and W35 showed that as the initial water content of SC increased, the pore space of the SGP-solidified soil increased and the production of flocculated hydration and gelled products decreased. Continuing to zoom in also revealed voids where the soil particles were in a state of dispersion from one another. This was because with the increase in initial water content of SC, the concentration of alkaline environment required for the hydration reaction of the SGP ternary binder decreased, resulting in a decrease in the dissolution degree of the SGP ternary binder particles, a weakening of the hydration reaction, a decrease in the number of hydration products and a weakening of the bonding between soil particles, resulting in the deterioration of the microstructure of the SGP-solidified soil.

Comparing the microscopic morphology of groups F1 and F2, it can be seen that the inter-particle connections between the soil particles of group F2 (mixed with 2% TEA) were more tightly packed, and the pores tended to change from large to small and from more to less. This was because TEA can play the role of electrical neutralization and adsorption bridging after dissolution, and it had an adsorption and complexation effect on the ions in solution, which promoted the ion migration and the continuous dissolution of reactive SiO_2_. Thus, the hydration degree of the SGP ternary binder was increased to generate more C(N)-A-S-H and C-S-H gels. These gels wrapped around the surface of the soil particles to fill the pores, and the gels were interspersed with each other, making the soil structure stable and dense. The strength of SGP-solidified soil was improved, which was consistent with the better UCS of F2.

In addition, some disclaimers and waivers needed to be made about the description of this section. The mechanisms described in this study were proposed based on current experimental results and interpretations of those results. These findings were revelatory rather than conclusive, and further research was needed to confirm the generalizability and validity of these mechanisms.

#### 3.5.3. EDS Spectrum Analysis

**Figure 12:**
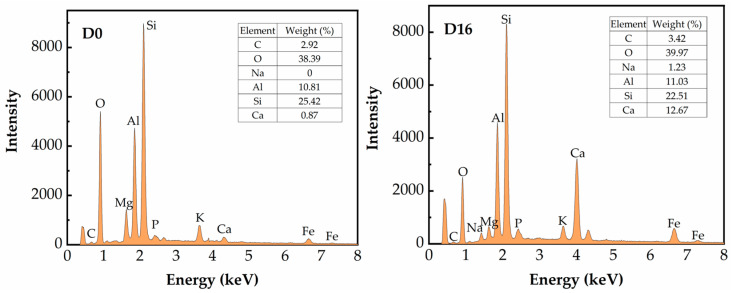
EDS test results of hydration products of D0 and D16.

Figure 12 displays a comparison of the EDS results of untreated and D16. By analyzing the difference between each constituent element of D16, the types of hydration products of the SGP-solidified soil can be qualitatively analyzed. The results showed that the gels of the SGP-solidified soil were mainly composed of Ca, Si, Al, Na, O and a small amount of Fe. C was present because, during the hydration reaction, the exciter was more basic, so it absorbed acidic gases from the air and produced carbonates to increase the carbon content of the system. The ratios of Si/Al, Ca/Si and Na/Al played an important role in the structure and rate of geopolymer generation, and the ratios of Si/Al, Ca/Si and Na/Al in the SGP-solidified soil specimens were 2.03, 0.56 and 0.11, respectively, which were in the reasonable range for the formation of geopolymer gels and geopolymerization [62,63]. A high elemental content of Al was also found, suggesting that some of the Si in the molecular chain was replaced by Al to form a C-A-S-H gel. Therefore, based on the above analysis, the hydration of the SGP-solidified soil was determined to be C-S-H and C(N)-A-S-H gels.

## 4. Solidification Mechanism of the SGP-Solidified Soil

The solidification mechanism of the SGP-solidified soil was mainly attributed to the geopolymerization and ion-exchange reactions of the SGP-solidified soil. Increasing the dosage of the SGP ternary binder, adjusting the initial water content of SC and mixing additives were conducive to increasing the degree of hydration of the SGP-solidified soil to form more gels, while the hydration products can promote the exchange of ions and enhance the agglomeration effect of the soil particles, thus improving the strength.

Geopolymerization reaction of the SGP-solidified soil: firstly, under the alkaline environment provided by Na_2_SiO_3_ and NaOH, the Si-O-T (T = Si or Al) of active SiO_2_ and Al_2_O_3_ in the SGP ternary binder and SC were broken, so that the silicon–oxygen tetrahedron and aluminum–oxygen tetrahedron were released into the system. Then, oligomers were formed through ionic polymerization and dehydration condensation. Meanwhile, under the excitation of the alkali activator, the content of Na^+^ ions in the system increased, and GBFS and PS released a large amount of Ca^2+^, which further recombined with the oligomers to generate gels such as hydrated C-S-H, N-A-S-H and C-A-S-H gels. Moreover, excess Ca^2+^ will combine with OH^−^ to produce Ca(OH)_2_ flake crystals, which will absorb CO_2_ from the air in an alkaline environment to carbonize to produce CaCO_3_ crystals. The Ca^2+^ in the system would replace part of the Na^+^ in the N-A-S-H gel product by ion exchange, thus promoting the conversion of N-A-S-H gels to C(N)-A-S-H gels. The gels generated by hydration bound the soil particles, filled the pores, enhanced the compactness of the soil and thus promoted the development of UCS.

Ion exchange reaction of the SGP ternary binder: Ca^2+^ dissolved from CaO in the SGP ternary binder was enriched on the surface of soil particles, replacing Na^+^ and K^+^ ions adsorbed on the surface of soil particles, reducing the thickness of the double electric layer, decreasing the repulsive force between soil particles and making the connection between soil particles more tightly connected, which promoted the agglomeration and flocculation of soil particles, ultimately decreasing the porosity of the soil body and optimizing the strength of the SGP-solidified soil.

## 5. Conclusions

In this paper, a novel SGP ternary binder was prepared by using SCCG, GBFS and PS. Firstly, alkali activator content, modulus of alkali activator and mass ratio of GBFS and PS were optimized by orthogonal experiments. Then the effects of the SGP ternary binder content, initial water content of SC and types of additives on the UCS of the SGP-solidified soil were analyzed. Finally, the solidification mechanism of the SGP ternary binder on SC was investigated by combining XRD analysis and SEM-EDS analysis. The main conclusions are shown below:

(1) The results of orthogonal experiments showed that the mass ratio of GBFS and PS was the main factor affecting the USC, and the alkali activator content and modulus of the alkali activator were the secondary factors, among which the modulus of the alkali activator had the least effect on the strength. The optimal combination of the SGP ternary binder was A_2_B_2_C_3_, i.e., the alkali activator content was 13%, the modulus of the alkali activator was 1.3 and GBFS:PS = 2:1. At this time, the 7 d and 28 d UCS were 2.048 MPa and 2.462 MPa, respectively.

(2) When the SGP ternary binder content was 16% and the initial water content of SC was 35%, the 28 d USC of the SGP-solidified soil reached up to 3.29 MPa, which met the requirements of the CJJ/T286-2018 “Technical Standards for Application of Soil Curing Agents” for the UCS of tertiary cured soil.

(3) The incorporation of TEA and PVC improved the UCS of the SGP-solidified soil, while the incorporation of Na_2_SO_4_ significantly deteriorated the UCS of the SGP-solidified soil. This was because the Na^+^ concentration in the SGP-solidified soil increased after Na_2_SO_4_ was incorporated, and after a large amount of Na^+^ was adsorbed on the surface of the negatively charged soil particles, the thickness of the soil particle bilayer became thicker, and the adhesion between the soil particles was weakened, which led to the structure of the SGP-solidified soil becoming loose. In contrast, the TEA and PVC were able to play the roles of electrical neutralization and adsorption bridging, with adsorption and complexation of ions in solution, which promoted the continuous dissolution and hydration of SiO_2_ and the improvement of UCS.

(4) From the results of XRD and SEM-EDS tests, it can be seen that the C-S-H gels and C(N)-A-S-H gels generated by the hydration of the SGP-solidified soil interpenetrate, intertwine and adhere to each other to form a network-like agglomeration structure, which was capable of filling the inter-pore spaces between the soil particles while cementing the soil particles, and the UCS of the SC was enhanced. The generation amount of hydration products, the degree of development, uniformity and the degree of soil densification together determined the size of the strength of cured soil.

## Figures and Tables

**Table 1:** Physical properties of SC.

Sample	Moisture Content	Liquid Limit	Plastic Limit	Plasticity Index	Specific Gravity
**Untreated SC**	47%	41.9%	22.3%	19.6	2.62 g/cm^3^

**Table 2:** Chemical properties of SC, SCCG, GBFS and PS.

Chemical Composition	SiO_2_	Al_2_O_3_	CaO	SO_3_	Fe_2_O_3_	P_2_O_5_	K_2_O	MgO	Na_2_O
**SC, %**	60.50	20.80	1.80	0.69	8.60	1.12	3.15	0.95	0.28
**SCCG, %**	64.20	21.69	3.27	0.27	4.66	0.81	0.11	2.83	0.45
**GBFS, %**	25.62	12.10	50.22	2.41	0.31	5.17	0.01	-	0.41
**PS, %**	39.05	3.99	47.16	0.72	2.07	2.94	1.99	-	-

**Table 3:** Orthogonal text-level factors.

Factors	Alkali Activator Content (A)	Modulus of Alkali Activator (B)	GBFS:PS (C)
**Level 1**	11%	1.1	1:2
**Level 2**	13%	1.3	1:1
**Level 3**	15%	1.5	2:1

**Table 4:** Mix proportion of the SGP-solidified soil.

Series	Sample	SGP Ternary Binder Content	Initial Water Content of SC	Additives
**D**	**D0**	0	35%	None
	**D13**	13%	35%	None
	**D16**	16%	35%	None
	**D19**	19%	35%	None
	**D22**	22%	35%	None
**W**	**W30**	16%	30%	None
	**W35**	16%	35%	None
	**W40**	16%	40%	None
	**W45**	16%	45%	None
**F**	**F1**	16%	35%	None
	**F2**	16%	35%	2% Triethanolamine (TEA)
	**F3**	16%	35%	2% Polyvinyl alcohol (PVA)
	**F4**	16%	35%	2% Na_2_SO_4_
	**F5**	16%	35%	1% TEA
	**F6**	16%	35%	3% TEA

**Table 5:** Orthogonal experiment results of the SGP-solidified soil.

Sample	Dosages of Activator (A)	Modules (B)	GBFS:PS (C)	7 d UCS	Standard Deviation
**O1**	1 (11%)	1 (1.1)	1 (1:2)	1.021	0.115
**O2**	1 (11%)	2 (1.3)	2 (1:1)	1.331	0.136
**O3**	1 (11%)	3 (1.5)	3 (2:1)	1.996	0.144
**O4**	2 (13%)	1 (1.1)	2 (1:1)	1.594	0.128
**O5**	2 (13%)	2 (1.3)	3 (2:1)	2.249	0.136
**O6**	2 (13%)	3 (1.5)	1 (1:2)	1.026	0.087
**O7**	3 (15%)	1 (1.1)	3 (2:1)	2.062	0.176
**O8**	3 (15%)	2 (1.3)	1 (1:2)	1.121	0.153
**O9**	3 (15%)	3 (1.5)	2 (1:1)	1.197	0.161

**Table 6:** Range analysis for 7 d unconfined compression strength results.

Curing Age	Factor	K_j1_	K_j2_	K_j3_	R_j_	Number of Levels	Number of Repetitions per Level	Importance Order
**7** **d**	A	4.438	4.869	4.38	0.174	3	3	C > A > B
	B	4.677	4.701	4.219	0.161	3	3	
	C	3.168	4.122	6.307	1.046	3	3	

## Data Availability

Data are contained within the article.
